# Feather barbs as a good source of mtDNA for bird species identification in forensic wildlife investigations

**DOI:** 10.1186/2041-2223-2-16

**Published:** 2011-07-28

**Authors:** Camilla F Speller, George P Nicholas, Dongya Y Yang

**Affiliations:** 1 Centre for Forensic Research, Ancient DNA Laboratory, Department of Archaeology, Simon Fraser University, 8888 University Drive, Burnaby, BC, V5A 1S6, Canada; 2 Department of Archaeology, University of Calgary, 2500 University Drive, Northwest Calgary, AB, T2N 1N4, Canada; 3 Department of Archaeology, Simon Fraser University, 8888 University Drive, Burnaby, BC, V5A 1S6, Canada

## Abstract

**Background:**

The ability to accurately identify bird species is crucial for wildlife law enforcement and bird-strike investigations. However, such identifications may be challenging when only partial or damaged feathers are available for analysis.

**Results:**

By applying vigorous contamination controls and sensitive PCR amplification protocols, we found that it was feasible to obtain accurate mitochondrial (mt)DNA-based species identification with as few as two feather barbs. This minimally destructive DNA approach was successfully used and tested on a variety of bird species, including North American wild turkey (*Meleagris gallopavo*), Canada goose (*Branta canadensis*), blue heron (*Ardea herodias*) and pygmy owl (*Glaucidium californicum*). The mtDNA was successfully obtained from 'fresh' feathers, historic museum specimens and archaeological samples, demonstrating the sensitivity and versatility of this technique.

**Conclusions:**

By applying appropriate contamination controls, sufficient quantities of mtDNA can be reliably recovered and analyzed from feather barbs. This previously overlooked substrate provides new opportunities for accurate DNA species identification when minimal feather samples are available for forensic analysis.

## Background

Accurate identification of bird species is crucial for wildlife law enforcement and other aspects of wildlife forensics. Currently, many birds and bird products (such as feathers) are protected under the US Migratory Bird Treaty (MBTA), the US Endangered Species Act (ESA) and the Convention on International Trade in Endangered Species (CITES). Identification of these protected species by law enforcement personnel may be challenging when only partial or damaged feathers are available for examination. Additionally, other criminal investigations, such as bird larceny, may also be contingent upon accurate species identification of bird feathers [1]. Although morphologically-based identifications are possible when feathers are complete and intact, they may be unfeasible when feathers have been modified, dyed or damaged. In such cases, DNA-based species identification techniques can be far more accurate.

Feathers are made up of a calamus (or basal quill), which extends into the rachis (or main shaft), which then supports the barbs [2]. Most current DNA-extraction techniques for feathers are focused on the calamus. DNA is typically isolated from calamus cells, requiring the destruction of 5 to 10 mm of the feather-shaft terminus [3-9]. Other studies have also successfully obtained DNA from the blood clot located in the superior umbilicus of the feather shaft [10]. Rawlence *et al*. [11] recently investigated the potential for DNA extraction from the feather barbs and rachis, and reported that the distal portion of the feather (that is, the rachis and barbs) retained mitochondrial (mt)DNA. However, their DNA-extraction methods required the destruction of the entire feather.

Consequently, most existing DNA-extraction protocols are not suitable for dealing with damaged, modified or antique/archaeological feathers that may be missing the terminal portion of feather shaft. Additionally, extraction methods that require the destruction of the entire feather are not desirable when testing crafted items and artifacts such as headdresses and fans, particularly those that may be culturally valued or historically prized.

Both animal and human hairs, which are similar to feather barbs in that they are composed of keratin [2], have proven to be extremely good sources of mtDNA, especially in ancient or forensic contexts [12-14]. Ancient and forensic DNA techniques are designed to target low quantities of degraded DNA in order to retrieve DNA from degraded or minute evidentiary samples. Previous forensic studies applied to human hairs have been successful at retrieving DNA from extremely small sample sizes (that is, single hairs) [15,16]. Therefore, the objectives of this study were to 1) determine the feasibility of extracting and amplifying suitable quantities of mtDNA from just a few feather barbs, rather than the whole feather; and 2) to test the reliability and sensitivity of the minimally destructive analytical technique.

DNA was initially extracted from four freshly collected feathers from North American wild turkey (*Meleagris gallopavo*) and Canada goose (*Branta canadensis*), using two different DNA-extraction techniques to test the feasibility of extracting and amplifying mtDNA. After a rigorous decontamination technique, mtDNA samples were first extracted from two and five barbs per feather using a modified silica-spin extraction protocol designed for degraded DNA samples (modified silica-spin column (MSSC) protocol) [17]. Subsequently, the mtDNA was extracted using a commonly available commercial DNA-extraction kit (see Methods). The DNA extracts were amplified by PCR, targeting fragments 200 to 300 bp in length of the mitochondrial DNA cytochrome *b *gene of various bird species (Table 1).

**Table 1:** Cytochrome b primers for PCR amplification of feather samples

**Primer**^**a**^	Sequences (5'→3')	Amplicon length, bp	Targeted taxa
EG-F10	CTAGGAATCTGCCTACTAACACAAA	297	Passeriformes
		
EG-R305	TCATGGGAGTACGTAGCCTACGA		

EG-F144	CGCCAATGGAGCATCCTTCTTC	219	Passeriformes
		
EG-R370	CAATGTAAGGGATGGCTGAGAATA		

GA-F407	GRGGRCAAATATCATTYTGAGG	220	Galliformes/ Anseriformes
		
GA-R627	GGRTTGTTTGAGCCYGATTCG		

GC-F108	CCTCCTCGGAATCTGCCTAAC	241	*Glaucidium *Sp.
		
GC-R349	CTGTGTTYCAGGTTTCTTTGTG		

AH-F122	GCCTAATGACACAAATCCTAACCG	200	*Ardea *Sp.
		
AH-R322	ACGAGCCGTAATAGAGTCCGC		

^a^F and R in the primer name denote forward and reverse primers, respectively.

**Figure 1:**
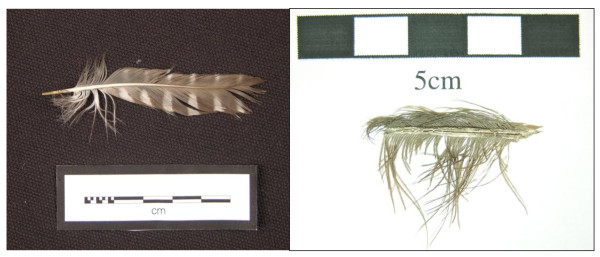
**Whole-feather samples**. **(Left) **Pygmy owl (*Glaucidium californicum*) feathers obtained from the SFU Department of Archaeology's zooarchaeological reference collection; **(right) **200-year-old magpie (*Pica pica*) feather recovered from archaeological site near Kamloops, BC.

Once the initial protocol had been tested on fresh feathers, the technique's reliability and sensitivity was tested using feathers of blue heron (*Ardea herodias*) and pygmy owl (*Glaucidium californicum*) (Figure 1a) five to 10 years old a museum-curated specimen of ruffed grouse (*Bonasa umbellus*) and an archaeological specimen of magpie (*Pica pica*) feather (Figure 1b).

## Results

### PCR amplification

**Figure 2:**
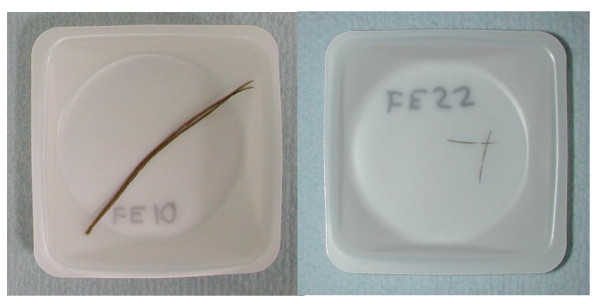
**Examples of two-barb samples used in analysis**. (Left) FE10 = Wild turkey; (right) FE22 = pygmy owl, Scale shown by weighing boat, which has an edge length of 40 mm.

PCR was performed for all feather samples, comprising two-barb and five-barb samples from the fresh, stored, museum and archaeological specimens (Figure 2; Table 2). Four feathers from wild turkey and Canada goose were extracted using two different protocols, MSSC and the Qiagen DNA Investigator Kit (QDIK) (see Methods). There was no difference in the success rates PCR amplification for the two DNA-extraction methods; both the MSSC protocol and the QDIK protocol yielded successful amplifications for both the two-barb and five-barb wild turkey and Canada goose feather samples.

**Table 2:** Results of PCR amplifications, sequencing and species identifications for two-barb and five-barb feather samples

Code	Sample	Barbs, n	Extraction method	Sequence quality	Species (Genbank accession number)
FE1	Wild turkey (fresh)	5	MSSC	Clear	*Meleagris gallopavo *(JF303667)
	
FE2	Wild turkey (fresh)	5	MSSC	Clear	
	
FE3	Wild turkey (fresh)	5	QDIK	Clear	
	
FE4	Wild turkey (fresh)	5	QDIK	Clear	

FE5	Canada goose (fresh)	5	MSSC	Clear	*Branta canadensis *(JF303668)
	
FE6	Canada goose (fresh)	5	MSSC	Clear	
	
FE7	Canada goose (fresh)-	5	QDIK	Clear	
	
FE8	Canada goose (fresh)	5	QDIK	Clear	

FE9	Wild turkey (fresh)	2	MSSC	Clear	*M. gallopavo *(JF303667)
	
FE10	Wild turkey (fresh)	2	MSSC	Clear	
	
FE11	Wild turkey (fresh)	2	QDIK	Clear	
	
FE12	Wild turkey (fresh)	2	QDIK	Clear	

FE13	Canada goose (fresh)	2	MSSC	Clear	*B. canadensis *(JF303668)
	
FE14	Canada goose (fresh)	2	MSSC	Clear	
	
FE15	Canada goose (fresh)	2	QDIK	Clear	
	
FE16	Canada goose (fresh)	2	QDIK	Clear	

FE17	Ruffled grouse (museum)	5	MSSC	Clear	*Bonasa umbellus *(JF303669)
	
FE18	Ruffled grouse (museum)	2	MSSC	Mixed peaks	

FE19	Blue heron (stored)	5	MSSC	Clear	*Ardea herodias *(JF303671)
	
FE20	Blue heron (stored)	2	MSSC	Clear	

FE21	Pygmy owl (stored)	5	MSSC	Clear	*Glaucidium californicum *(JF303670)
	
FE22	Pygmy owl (stored)	2	MSSC	Clear	

EG1	Magpie (archaeological)	2	MSSC	Clear	*Pica pica *(JF303672)
	
EG2	Magpie (archaeological)	5	MSSC	Clear	

**Figure 3:**
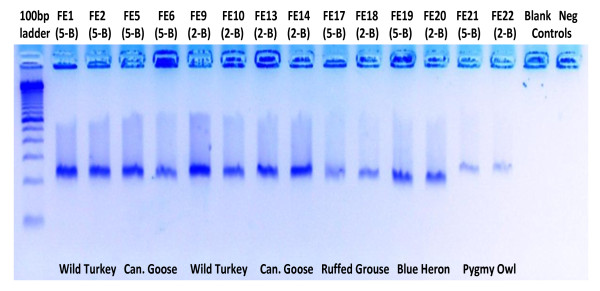
**Electrophoresis gel of cytochrome b fragments from feather specimens amplified by PCR with the MSSC extraction protocol**. FE# indicates individual feather specimens, 5-B and 2-B indicate five-barb and two-barb feather samples, respectively. 100 bp ladder is from Invitrogen (Carslbad, CA, USA).

Based on visual analysis of the electrophoresis gels, no differences were seen in the amplification strengths of the two-barb samples versus the five-barb samples for the fresh, stored and museum samples using either the MSSC extraction protocol (Figure 3) or the QDIK extraction protocol (data not shown). Only for the archaeological magpie feather did extracts from five-barb samples yield stronger amplification and longer fragments than extracts from two-barb samples.

### DNA sequencing and species identification

**Figure 4:**
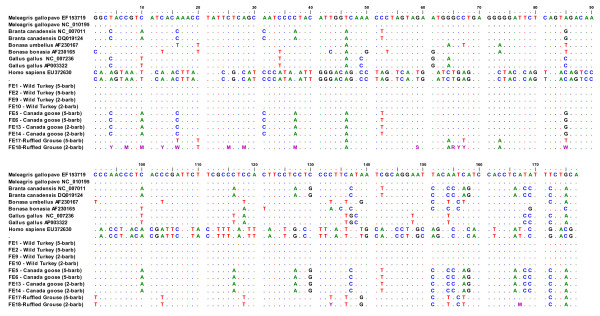
**Multiple alignment of cytochrome b sequences from feathers of wild turkey, Canada goose and ruffed grouse**. Dots indicate base pairs that are identical to the reference sequence (*Meleagris gallopavo *EF153719). In all but one case, the obtained sequences were either identical to or one base-pair different from the published reference sequences of the known species obtained from GenBank (accession numbers listed). Sample FE18, the two-barb grouse sequence, displayed ambiguous bases, probably caused by low template and potential contamination from chicken DNA.

In all cases in which clear sequences were recovered, DNA sequences were either identical to or very similar to the published GenBank reference sequences for all the tested species (Table 2; Figure 4). The reliability of the species identifications was supported by the match between the known species identity and the obtained sequences in 23 of the 24 samples (Table 2). Furthermore, all PCR reactions from the same feather yielded identical sequences, supporting the authenticity of the obtained sequences.

Clear sequencing results were obtained from 23 of the 24 samples, and no heteroplasmy was present in any of the samples. Among those 23 samples, no differences were seen in the sequencing quality of the two-barb compared with the five-barb samples for the fresh, stored and archaeological feather samples; there were no differences in sequencing quality between the MSSC and the QDIK extraction protocols.

Only one sample exhibited poor sequencing quality; sample FE18, the two-barb sample of the museum-curated ruffed grouse, displayed mixed peaks in the electropherogram, although good sequence quality was seen in the five-barb sample from the same feather. Upon further analysis, the mixed peaks visible in the electropherogram of the FE18 suggested that template problems (low quality and/or low quantity) and exogenous DNA from the domestic chicken (*Gallus gallus*) might have resulted in the poor sequence quality (Figure 4). The small amount (only two feather barbs) and antiquity of the samples (over 50 years old) probably resulted in a low quantity of authentic DNA template, which may have been overwhelmed by exogenous DNA during the amplification process.

## Discussion

### Reliability and sensitivity of feather-barb analysis

The amplification of mtDNA from all 24 feather samples, including fresh, stored, museum and archaeological samples, clearly demonstrates the feasibility of recovering mtDNA from just a few feather barbs, rather than the whole feather. The mtDNA amplification and sequencing yielded confident species identification from as little as two barbs, indicating that feathers represent an excellent substrate for minimally destructive DNA techniques. Moreover, the recovery of mtDNA using two different extraction protocols indicates that feather barbs are a reliable DNA substrate.

The generally clear sequencing results and reliable species identifications obtained using both the two-barb and five-barb samples indicate that even a few feather barbs are suitable for mtDNA-based species identification analyses. However, the poor sequencing quality and the presence of mixed peaks visible in the electropherogram of the museum-curated ruffed-grouse specimen highlights the high potential for contamination of low-template DNA samples by exogenous sources.

No chicken DNA had been processed in the Forensic DNA Laboratory at Simon Fraser University (SFU), suggesting that sample cross-contamination was not the source of any exogenous DNA in the two-barb grouse sample. However, the sporadic presence of animal DNA in laboratory reagents has been noted for several years, especially for common domestic animals such as chicken, cattle and pig [18]. Forensic DNA extraction and amplification techniques are designed to target minute amounts of evidentiary DNA, and thus even very low concentrations of contaminant animal DNA can produce false-positive results [19]. Although the low quantities of animal DNA in laboratory reagents may not be an issue for human DNA studies, they may pose a challenge to wildlife forensic investigations. Therefore, the results from this study highlight the need for both carefully designed primers and for dedicated laboratory workspaces when analyzing of low-template DNA.

Primers can be designed specifically to minimize the potential for contamination from modern sources. When working with trace wildlife forensic samples or low-template extracts, taxon-specific primers are recommended to exclude contamination both from modern human sources and from common exogenous animal species.

The results of this study also highlight the need for a thorough sample-decontamination process and the use of a clean DNA-extraction laboratory when applying these minimally destructive analytical techniques, particularly when working with feather samples several decades old. Moreover, when working with degraded or ancient feathers, a larger sample may need to be extracted (for example, five to six feather barbs) to ensure adequate quantities of DNA template for amplification.

Interestingly, no evidence of contamination (for example, mixed electropherogram peaks, amplification of blank extracts, or negative controls) were seen in the archaeological magpie feather samples processed in the Ancient DNA Laboratory at SFU. This laboratory is dedicated to the DNA analysis of archaeological samples that are often several thousands of years old, and thus practices extremely rigorous contamination prevention controls, which seem to have been effective in eliminating any systematic laboratory contamination.

### Applications and contributions

Minimally destructive DNA-extraction techniques are extremely beneficial in forensic contexts, as evidentiary feather samples may be left virtually intact for subsequent morphological studies. Additionally, the results of this analysis indicate that DNA can be successfully retrieved from feather barbs using a commonly available commercial DNA-analysis kit, making this technique easily implementable in most forensic laboratories.

Minimally destructive DNA techniques for feather barbs have applications in a variety of national and international forensic contexts. Such analyses will benefit wildlife law enforcement, which is responsible for identifying the illegal possession of feathers (and other bird products) from species protected under the MBTA, ESA and CITES. This minimally destructive technique will be valuable for identifying crafted trade products or artifacts incorporating the feathers of protected birds. Furthermore, this highly sensitive technique can be used on small, damaged or degraded feathers in other forensic contexts, including the identification of species involved in bird strikes (collision between birds and transport vehicles, usually aircraft) or hazardous environmental incidents (for example, oil spills).

In addition to forensic contexts, minimally destructive DNA techniques for bird feathers may benefit several other fields, including conservation biology and archaeology. Such DNA-extraction techniques may be applied to non-invasively collected bird feathers and/or museum samples of threatened, extinct or extirpated bird populations, thus benefiting ornithologists and conservation biologists. Likewise, this study demonstrates the feasibility of using a few feather barbs for identifying bird species used in archaeological contexts.

## Conclusions

In this study, we have shown that bird feather barbs are a good source of mtDNA. Furthermore, we found that, using appropriate contamination controls, mtDNA can be recovered and reliably analyzed from as few as two feather barbs from both fresh and degraded feathers, leaving the remainder of the feather intact. This study highlights the suitability of feather barbs as a minimally destructive source of DNA for forensic wildlife cases, and for DNA analysis of rare museum and/or archaeological feather samples.

## Methods

### Feather samples

DNA was extracted from eight feather specimens of known species with various degrees of degradation to test the feasibility and sensitivity of the developed protocol. DNA extraction was first tested on four freshly collected feathers from North American wild turkey (*M. gallopavo*) and Canada goose (*B. canadensis*) (Table 2). The fresh feather specimens used in this study were shed feathers from morphologically identified wild birds, rather than from voucher specimens [20]. Once the initial protocol had been tested, the technique's reliability and sensitivity was tested using feathers of blue heron (*A. herodias*) and pygmy owl (*G. californicum*) (both 5 to 10 years old), obtained from the zooarchaeological reference collection of the Department of Archaeology at SFU (Figure 1, left image). Finally, DNA analysis was also applied to much older feather samples in order to test the sensitivity of the technique and its suitability for degraded feathers. The degraded samples were a museum-curated specimen, about 50 years old, of ruffed grouse (*B. umbellus*), obtained from the Prince of Wales Heritage Centre in Yellowknife, NWT, and a magpie (*P. pica*) feather, about 200 years old, recovered from archaeological site EeRb-144, located near Kamloops, BC (Figure 1, right image).

### Sample preparation

Sample preparation and DNA extraction of the fresh and museum-curated feather samples were conducted in the Forensic DNA Laboratory in the Centre for Forensic Research at SFU. This laboratory follows comprehensive controls recommended for the analysis of human and non-human animal forensic remains [21,22], including: positive pressure air flow, use of dedicated equipment including clothing, equipment and reagents, and inclusion of multiple blank extracts and negative controls through every stage of analysis. DNA extraction of the archaeological feather followed the same protocols as the fresh samples, but was conducted in the Ancient DNA Laboratory at SFU, which is dedicated to the analysis of samples more than 100 years old. Similarly, this laboratory follows strict contamination-control protocols for the analysis of ancient remains [23,24].

For all eight feathers, two and five feather barbs were removed from the feather shaft using sterile scissors or a sterile scalpel blade (Figure 2), resulting in a total of 24 tested samples (Table 2). For all 24 samples, barbs were first rinsed in 3% sodium hypochlorite for 30 seconds to remove possible surface contamination, then rinsed twice in DNase/RNase-free distilled water (UltraPure™, Invitrogen, Carlsbad, CA, USA). After decontamination, two different DNA-extraction techniques were applied.

### DNA extraction

The samples first underwent an MSSC extraction protocol designed for degraded DNA samples [17]. As keratin is the major structural component of feather barbs, we modified the lysis buffer to include dithiothreitol (DTT). Barbs were incubated in 1.5 to 3 ml of lysis buffer (0.05 mol/l EDTA pH 8.0, 0.5% SDS, 0.5 mg/ml proteinase K and 10 mg/ml DTT) at 55°C for 1 to 2 hours. The remainder of the DNA extraction followed our previously described protocol [23], producing a final elution of 100 μl of DNA solution for each sample.

Subsequently, the feather-barb samples of wild turkey and Canada goose were also extracted using a commercial kit (Qiagen's DNA Investigator Kit; Qiagen, Valencia, CA, USA) (QDIK protocol), following the manufacturer's protocol designed for hair, producing a final elution of 100 μl of DNA solution for each sample.

### PCR amplification

Primers were designed to target fragments 200 to 300 bp in size of the mitochondrial DNA cytochrome *b *gene of various bird species (Table 1). The cytochrome *b *gene was specifically targeted because this locus displays sufficient sequence diversity to distinguish species, yet relatively low levels of intraspecies variation [25]. The cytochrome b gene is commonly used in both wildlife forensic and phylogenetic contexts when robust species identification is required [20,26,27]. When designing the primers, special consideration was given to the specificity of the targeted PCR products. Using BLAST searches and multiple alignments of numerous reference sequences downloaded from GenBank, the primers sets were carefully designed to separate the bird species used in this study.

PCR was conducted in a Mastercycler (Eppendorf, Westbury, NY, USA) in 30 μl PCR reactions containing 50 mmol/l KCl, 10 mmol/l Tris-HCl, 2.5 mmol/l MgCl_2_, 0.2 mmol/l dNTP, 0.3 μmol/l primer, 1.5 mg/ml BSA, 3 μl of DNA sample and 0.75 U AmpliTaq Gold™ (Applied Biosystems, Carlsbad, CA, USA). PCR was conducted with an initial denaturing step at 95°C for 12 minutes, followed by 35 to 40 cycles at 94°C for 30 seconds and 55°C for 30 seconds, and a final extension step at 72°C for 40 seconds. DNA samples (5 μl) were separated by electrophoresis and visualized through a dark reader (Clare Chemical Research Co., USA). Successfully amplified products were purified using a commercial kit (MinElute™PCR Purification Kit (Qiagen) or ExoSAP-IT^® ^(USB Corporation, Santa Clara, CA, USA)). PCR products were sequenced using both forward and reverse primers either at the Macrogen Inc. sequencing facility in (Seoul, Korea) or the Eurofins MWG Operon sequencing facility (Huntsville, AL, USA).

### DNA sequencing and species identification

DNA sequences were visually edited, and base-pair ambiguities were examined using ChromasPro software http://www.technelysium.com.au and compared with modern published references through the GenBank BLAST application http://www.ncbi.nlm.nih.gov/BLAST/. Multiple alignments of the obtained sequences and published bird reference sequences were conducted using ClustalW [28], through BioEdit http://www.mbio.ncsu.edu/BioEdit. Species identifications were assigned to a sample only if it was identical to or very similar to the published reference sequences [29-32], and if no other evidence, including reproducibility tests or additional sequencing of the same sample, indicated a different species.

Accession numbers:

[GenBank: JF303667-JF303672]

## Abbreviations

BLAST: Basic Local Alignment Search Tool; EDTA: ethylene diamine tetraacetic acid; PCR: polymerase chain reaction; MSSC: modified silica-spin column; QDIK: Qiagen's DNA Investigator Kit; SFU: Simon Fraser University.

## Competing interests

The authors declare that they have no competing interests.

## Authors' contributions

CFS participated in the design of the study, performed DNA extractions and PCR amplifications, conducted sequence alignments and species identifications, and drafted the manuscript. GPN conceived of the study, participated in its design and helped to draft the manuscript. DYY participated in the design and coordination of the study, performed DNA extractions and amplifications, and helped to draft the manuscript. All authors read and approved the final manuscript.
